# Opportunities and Challenges in Using Artificial Intelligence in Guideline Development and Implementation

**DOI:** 10.1002/gin2.70029

**Published:** 2025-08-25

**Authors:** Chirine Chehab, Xiaomei Yao, Jonathan L. Heald, Stacy Lathrop, Heba Hussein, Maria Michaels

**Affiliations:** ^1^ Centers for Disease Control and Prevention (CDC) Atlanta Georgia USA; ^2^ Department of Health Research Methods Evidence and Impact McMaster University Hamilton Ontario Canada; ^3^ Program in Evidence‐Based Care, Department of Oncology McMaster University Hamilton Ontario Canada; ^4^ The Infectious Diseases Society of America Arlington Virginia USA; ^5^ National Center for Biotechnology Information, National Library of Medicine (NLM), National Institutes of Health (NIH) Bethesda Maryland USA; ^6^ Department of Oral Medicine and Periodontology Faculty of Dentistry Cairo University Cairo Egypt

## 1 Introduction

Artificial intelligence (AI) within clinical care is a burgeoning area of interest. AI is rapidly transforming the landscape of clinical care, revolutionising how healthcare is delivered, managed and optimised [1]. With its ability to analyse vast amounts of data, identify patterns and make real‐time predictions, AI holds immense promise in improving patient outcomes, enhancing efficiency and reducing healthcare costs when the technology is used responsibly and its potential risks are mitigated. AI tools are currently being explored for training clinicians, enhancing the rigour, breadth and precision of environmental and biomedical health data, and reporting, evaluating and synthesising data in biomedical research and clinical practice [2, 3]. In response, biomedical research and public health agencies are developing frameworks and guidelines for the use of AI in clinical research and direct patient care [1, 4, 5]. Similarly, funding agencies, scholarly editors and shared oversight bodies are releasing advisories and guidelines for investigators and authors engaged in peer review and writing [6, 7].

Clinical Practice Guidelines (CPGs) are cornerstones of evidence‐based medicine, providing standardised recommendations for patient care [8]. As part of ‘Bringing Guidelines to the Digital Age’, an effort by the Guidelines International Network North America (GIN‐NA) regional community that applied human‐centred design (HCD) to solve pain points in guideline development and implementation, a multidisciplinary team of systematic reviewers, guideline developers, informaticists, and others worked collaboratively to gain insights, conduct research, and identify possible solutions for three pain points in guideline development and implementation [9, 10]. The pain points included (1) insufficient informatics resources and expertise to translate ambiguous or complex language in written guidelines into computable formats, (2) incomplete information in existing guidelines that necessitates adapting guidelines to cater for the needs of diverse patient populations, and (3) unclear language, which creates barriers for patients and clinicians to understand and use guidelines [9].

Given the widespread availability of AI tools, the team emphasised the need to help guideline developers understand how approaches that include AI tools could address these challenges and facilitate guideline development and implementation. These pain points serve as a framework for exploring potential AI applications within each stage of guideline development. Through weekly meetings, the team looked at potential areas for automation with AI tools in the life cycle of guideline development, adapted from the GIN‐McMaster Guideline Development Checklist and the 2011 Institute of Medicine Report, Clinical Practice Guidelines We Can Trust [8, 10]. These automation approaches require a shift in behaviours and mindsets among guideline developers to effectively integrate AI tools into guideline development workflows.

Amid the rapid evolution of AI technology, we anticipate that enhanced AI tools with accuracy, validation and adherence to governance frameworks will facilitate the effective development of guidelines without significantly compromising quality. These tools could eventually surpass human performance in certain steps in the near future, but their use must remain grounded in principles of transparency, equity and accountability to fully realise their potential while upholding scientific integrity and public trust [11, 12]. The primary objective of this manuscript is to enlighten guideline developers to re‐evaluate current methods and leverage AI's power effectively within the CPG development and implementation continuum. By structuring our discussion to first explore AI's transformative potential, we aim to provide a comprehensive and practical perspective on its role in shaping the future of clinical guidelines, with a later section dedicated to addressing its risks and challenges.

## 2 Guideline Development

### 2.1 Evidence Synthesis and Moving From Evidence to Recommendations

AI tools could assist guideline developers in synthesising evidence from various sources, including clinical trials, systematic reviews, and observational studies [13]. Guideline developers are the gatekeepers in accepting and implementing evidence; however, they are increasingly grappling with the challenge of keeping systematic reviews and guideline recommendations up‐to‐date due to the rapid influx of new research and growing complexities of clinical practice [11].

Systematic reviews begin with formulating structured research questions, such as those framed using the Population, Intervention, Comparator and Outcome (PICO) format. After selecting the appropriate literature search databases, AI tools could generate search queries based on PICO questions that include text, numbers and sometimes special characters for each search database, such as PubMed or aggregated datasets like a registry or local healthcare data, collected from multiple sources in cloud‐based storage systems [14]. A recent study demonstrated how AI‐supported systems could help formulate guideline questions, enabling a more structured approach to evidence synthesis and decision‐making [15]. This study employed a large language model (LLM) to directly generate guideline questions. This approach resulted in the creation of 22 unique relevant questions, with 11 not previously suggested by the panel. Among the 11 questions generated solely via the LLM, 4 questions were prioritised by the panel, demonstrating how LLMs can enhance the diversity and relevance of questions considered in guideline development [15].

AI‐driven tools are increasingly being leveraged to streamline systematic review processes, a crucial step in evidence‐based guideline development. AI tools could detect duplicate publications by searching a database or using programmed queries. Following machine learning and training procedures by systematic reviewers, AI tools could screen titles and abstracts, obtain and screen the full text of the relevant articles and subsequently extract data from the included articles [14]. For instance, AI tools like RobotReviewer have demonstrated potential in expediting systematic review processes by screening abstracts and extracting data [12, 14]. They could analyse data and perform meta‐analyses. AI tools could then assess the risk of bias and certainty of evidence for each outcome per research question [5, 13].

AI tools could also assess contextual and personal queryable data from real‐time observations of patterns in available iteratively standardised datasets [12]. AI tools could standardise the transition from evidence to recommendations such as prioritising clinical questions, incorporating patient values, and identifying decisional factors like benefits and harms [15]. For example, natural language processing (NLP) tools may analyse patient preference data to guide recommendations [2]. If needed, AI tools could generate parts of the final report and its subsequent updates or living CPGs required from guideline developers. AI can support living systematic reviews by continuously monitoring and integrating new evidence [5, 13]. This ensures guidelines remain current in dynamic clinical environments. However, challenges include validating AI algorithms to ensure they adapt appropriately to changes in evidence and clinical contexts [4, 5].

Although there is currently no perfect AI tool to support guideline developers at every step described above, a few AI tools have been piloted within certain steps and achieved impressive time savings [12, 16]. For example, Clark et al. completed a systematic review, involving 1381 literature search result papers after deduplication and ultimately including eight papers, in 61 person‐hours (equivalent to 9 workdays) with the aid of AI tools [17]. However, they did not validate the precision of each step through human reviewers, underscoring the importance of human oversight in ensuring transparency and maintaining trust in the process.

By accelerating tasks across the systematic review and recommendations writing lifecycle, AI could potentially slow rising costs and transform guideline development and implementation, promising considerable efficiency and quality gains for guidelines [12]. One key advantage is AI systems' ability to capture vast amounts of data quickly and consistently. By automating tasks that are not inherently complex but are highly time‐consuming, guideline developers could redirect their attention towards more intricate responsibilities, thus optimising the utilisation [12].

Despite these advancements, ethical challenges remain, particularly concerning transparency, algorithmic bias and data privacy. Governance frameworks, such as the National Institute of Standards and Technology (NIST) AI Risk Management Framework (AI RMF) and the Ethics Guidelines for Trustworthy AI, can provide essential guidance in managing these risks while promoting equitable and ethical practices. Proactive collaboration among stakeholders, including guideline developers, healthcare professionals, and informaticists, is essential to establish robust validation processes for AI tools and ensure they meet acceptable standards of accuracy and fairness [1, 4].

## 3 Guideline Evaluation and Continuous Improvement

### 3.1 Real‐World Data Monitoring

AI could play a crucial role in real‐world data (RWD) monitoring in clinical guideline development and implementation. Increasing availability of RWD and rapid advancements in AI techniques, combined with the growing costs and limitations of conventional clinical trials, have generated significant interest in utilising RWD to improve the efficiency of clinical research and bridge the gap between evidence and practice, including supporting living guidelines [5, 18]. AI‐driven analysis of RWD is integral to the full life cycle of guideline development and implementation. RWD, sourced from electronic health records (EHRs), claims databases, patient registries, wearable devices and other data sources, allows for continuous assessment of guideline adherence, effectiveness and areas requiring refinement [19]. AI can also identify variations in clinical practice, detect unintended consequences of guideline adoption and provide real‐time insights into implementation barriers.

By analysing RWD, AI helps inform guideline updates, ensuring that recommendations remain responsive to evolving evidence and practice patterns. This data‐driven approach enables guideline developers to evaluate the impact of their recommendations and refine them based on emerging trends, ultimately enhancing the relevance and applicability of guidelines in diverse healthcare settings [19, 20]. AI could automate the collection and aggregation of RWD. For example, during the COVID‐19 pandemic, RWD supported the creation of real‐world evidence (RWE) concerning the virus's spread, treatment effectiveness, and the efficacy of COVID‐19 vaccinations. If we also apply AI to these and similar RWD/RWE efforts, we can enhance the precision and adaptability of guidelines [21].

RWD was leveraged to model localised strategies for controlling the spread of COVID‐19, analysing COVID‐19 and influenza using data from smartphones and wearables, investigating changes in behavioural and mental health linked to public life lockdowns and aiding in decision‐making and policy development, among other applications [21, 22]. By analysing data on infection rates, demographics and healthcare resource availability, public health officials could tailor their guidance and responses to the specific needs of different communities. This type of RWD integration would allow guideline developers to refine recommendations in real time, ensuring they remain responsive to emerging challenges and diverse population needs. However, AI algorithms must be validated and continuously evaluated to ensure they function appropriately across various types of data and for different groups of people to provide evidence of quality improvements and optimisation of resources [23]. The lack of standardised validation methods raises concerns about the reliability of AI‐generated evidence, necessitating rigorous quality control mechanisms.

In a prior investigation regarding the perspectives of guideline developers, the recommendation to employ a broader evidence foundation in the development and adaptation of guidelines was proposed to narrow the existing gap and to identify a patient's risk of developing a disease, which is particularly pertinent for specific patient cohorts that require targeted treatment; such analysis cannot be captured from clinical trials [18]. For instance, RWD enables the identification of patient subgroups with unmet needs, such as individuals at higher risk for adverse events or those who may benefit from specific interventions that clinical trials alone may not sufficiently address. AI algorithms could be tailored to identify trends and outliers that support the prediction of the risk of specific health outcomes or complications and the detection of adverse events and noncompliance that allow for a timely revision or intervention [23].

## 4 Guideline Review and Validation

### 4.1 Personalisation of Care

One of the limitations of most guidelines is that they may not include guidance for certain populations and do not take other patient‐specific factors into account. AI could enable the personalisation of care by analysing patient data, preferences and clinical characteristics to tailor treatment plans and guideline recommendations to individual needs [24, 25]. By integrating data from EHRs, wearable devices and other sources, AI could help identify undefined or underserved populations by analysing demographic, socioeconomic and health data to uncover disparities in access to care and health outcomes, recognising areas where guidelines have not sufficiently been applied [26].

For example, the Protocol for Responding to and Assessing Patients' Assets, Risks, and Experiences (PRAPARE) is a national effort to collect and apply data about social drivers of health. When paired with AI, PRAPARE enables health centres to tailor guidelines to address individual and community needs effectively [27]. Another tool, IBM's Watson for Oncology, has been piloted in assisting oncologists by analysing large datasets and providing treatment options aligned with patient preferences and clinical evidence [28]. These examples demonstrate the role of AI‐driven clinical decision support (CDS) tools in integrating real‐time recommendations into EHRs, streamlining decision‐making processes to improve patient outcomes and satisfaction [26]. AI could contribute to personalised care by considering individual patient characteristics, medical history, preferences and goals when implementing guidelines. This approach fosters shared decision‐making, empowering patients to participate actively in their healthcare, resulting in more tailored and effective treatment plans.

Furthermore, predictive analytics supported by machine learning algorithms could identify patients at high risk of adverse events. For example, an AI algorithm developed by Rajkomar et al. uses hospital EHR data to predict patient readmissions and mortality with high accuracy [29]. Such insights allow clinicians to proactively implement interventions, including closer monitoring or more aggressive treatment, thereby ensuring that clinical guidelines are applied in a manner that reflects patient‐specific risks and preferences [30].

## 5 Guideline Implementation

### 5.1 Use of Health Data Standards

Guideline developers can leverage AI in several ways to implement health data standards effectively, such as Fast Healthcare Interoperability Resources (FHIR), specifically Evidence‐Based Medicine on FHIR (EBM‐on‐FHIR) and FHIR Clinical Guidelines (CPG‐on‐FHIR) [31, 32]. FHIR defines how healthcare information can be represented consistently and exchanged between different computer systems regardless of how it is stored in those systems [33, 34]. EBM‐on‐FHIR and CPG‐on‐FHIR define how to use FHIR to represent, exchange, and perform functions related to computable evidence and guidelines. AI can support the rapid bidirectional translation of observational evidence in FHIR from healthcare systems to narratives formatted in scholarly communications standards for review by experts [32]. Critical portions of evidence and guidance may be modularly structured with persistent identifiers for FAIR (findable, accessible, interoperable, reusable) use through indexes, like PubMed, facilitating the ease of review and updating [35].

Furthermore, data can be iteratively curated with support from AI if structured in standardised vocabularies and elements [34]. Additionally, AI algorithms may enhance data security, privacy protection and compliance with regulatory frameworks, mitigating risks associated with data breaches and unauthorised access [1]. This capability reinforces trust among guideline developers, healthcare providers, and patients, while enabling seamless integration with systems designed to uphold international health data standards.

### 5.2 Challenges and Potential Dangers

AI presents both challenges and opportunities in guideline development and implementation, and it is crucial to acknowledge potential dangers, especially in sensitive areas like healthcare [34]. Rapid advances in LLMs like Bidirectional Encoder Representations from Transformers (BERT), Bidirectional and Auto‐Regressive Transformers (BART), and Chat Generative Pretrained Transformer (ChatGPT) have amazed the world with their ability to generate impressive texts, draft recommendations, summarise evidence, and surpass human performance in particular tasks [25, 26, 27, 30].

However, these AI models are also plagued by issues, including concerns related to algorithmic bias, data privacy, transparency and hallucinations, thus prompting the need for human oversight in AI‐driven decision‐making processes [16, 33]. For example, an Optum health care AI algorithm disproportionately favoured healthier white patients over sicker Black patients. The bias occurred because the algorithm relied on healthcare costs as a proxy for health needs, which led to underestimating the needs of Black patients, as they typically incur lower healthcare costs due to systemic inequities. This flaw resulted in Black patients being ranked lower in need for care management, even when they had more severe health conditions compared to their white counterparts, underscoring the need for rigorous validation and oversight in AI deployment [36].

Challenges such as algorithmic bias and data privacy remain critical considerations, necessitating robust validation and ethical oversight in the deployment of these technologies [4]. The black‐box nature of many AI models presents a challenge for guideline developers who need to justify recommendations. Without transparent methodologies, AI‐generated insights may be difficult to interpret, limiting their practical application in guideline formulation. Additionally, data privacy and transparency are significant concerns, particularly when AI models rely on large datasets that may contain sensitive patient information. Ensuring that these models adhere to strict privacy standards while remaining transparent about how decisions are made is essential to maintaining trust and compliance with regulations like the 1996 Health Insurance Portability and Accountability Act (HIPAA) in the United States [25].

Additionally, health equity concerns remain paramount as AI tools often perform less effectively for underrepresented groups due to biased training data. Addressing these disparities requires a commitment to inclusive and representative datasets. Hallucinations, errors in which AI models generate inaccurate or fabricated information, represent another critical danger. AI hallucinations can be particularly problematic in medical decision‐making, as even a slight misstep can lead to inappropriate treatment plans. This further highlights the importance of integrating AI with human oversight to catch errors before they impact patient care [37, 38].

The successful adoption of AI in guideline development may require investment in infrastructure, understanding sources of bias, training and governance frameworks to ensure responsible and ethical use of AI technologies. Frameworks and checklists such as NIST AI RMF, the Translational Evaluation of Healthcare AI (TEHAI) and the Consolidated Standards of Reporting Trials‐Artificial Intelligence (CONSORT‐AI) provide actionable guidance for ensuring responsible and ethical use of AI technologies in healthcare [1, 22, 37, 38]. For example, employing the prediction model risk of bias assessment tool (PROBAST), a tool for assessing prediction model risk of bias, can guide developers in validating AI algorithms before clinical implementation [22]. By focusing on these challenges, stakeholders can proactively address the risks associated with AI adoption in guideline development and implementation, ensuring its use enhances rather than undermines healthcare equity, accuracy and patient safety.

## 6 Conclusion

AI presents transformative opportunities to enhance evidence synthesis, guideline development, RWD monitoring, personalisation of care, and compliance with health data standards. By leveraging AI technologies responsibly and ethically, guideline developers could improve the relevance, effectiveness and accessibility of CPGs, maintaining scientific integrity with a patient‐centred approach considering individual variability and preferences, ultimately advancing evidence‐based healthcare and improving patient outcomes. Addressing challenges and potential dangers associated with AI adoption requires proactive measures, collaboration across stakeholders, and a commitment to upholding ethical standards in healthcare innovation. By adhering to governance frameworks and fostering collaboration, stakeholders can ensure that AI is leveraged responsibly, with an emphasis on transparency, equity, and patient‐centred care, thereby minimising ethical challenges and maximising transformative opportunities. Ongoing research should focus on validating AI tools for diverse clinical contexts, developing ethical frameworks for AI integration and ensuring transparency in AI‐supported decision‐making processes.

## Author Contributions

**Chirine Chehab:** conceptualisation, investigation, writing – original draft, methodology, validation, visualisation, writing – review & editing, formal analysis, project administration, data curation, supervision, resources, software. **Xiaomei Yao:** investigation, writing – original draft, validation, visualisation, writing – review & editing, formal analysis, data curation, resources. **Jonathan L. Heald:** investigation, writing – review & editing, visualisation, validation, formal analysis, data curation. **Stacy Lathrop:** investigation, visualisation, validation, writing – review & editing, formal analysis, data curation, writing – original draft. **Heba Hussein:** investigation, validation, visualisation, formal analysis, data curation. **Maria Michaels:** conceptualisation, investigation, funding acquisition, writing – original draft, methodology, validation, visualisation, writing – review & editing, formal analysis, data curation, project administration, resources.

## Disclosure

The findings and conclusions in this manuscript are those of the authors and do not necessarily represent the official position of the US Centers for Disease Control and Prevention or the National Institutes of Health. The Guidelines International Network (GIN; www.g-i-n.net) is a Scottish Charity, recognised under Scottish Charity Number SC034047. The views expressed in this article are those of the authors, and GIN is not liable for any use that may be made of the information presented.

## Conflicts of Interest

The authors declare no conflicts of interest.

## Data Availability

The authors have nothing to report.
